# Supplementary material for: Integrating a New Dietetic Care Process in a Health Information System: A System and Process Analysis and Assessment
Source: Int J Environ Res Public Health. 2022 Feb 22;19(5):2491. doi: 10.3390/ijerph19052491 (PMC8909013; doi:10.3390/ijerph19052491)
Supplement: Supplementary file 1 [file ijerph-19-02491-s001.zip › ijerph-1533807-supplementary/Supplementary Material S1.pdf]

Integrating a new dietetic care process (DCP) in a health information system (HIS): a system and process analysis and assessment

Gabriele Gäbler, Deborah Lycett and Walter Gall

Description of the new DCP

Results of the process analysis and assessment of the target process in regard of its sub-processes and document templates

1. Dietetics Assessment

The Dietetics Assessment as first sub-process of the DCP aims to collect and assess information and data on the nutritional status [62]. The required data are divided into three areas according to process- and information-technological aspects of the target process: 1. Administrative patient data, 2. Medical patient data, 3. Specific nutrition-related assessment data. The HIS already contains the administrative and medical patient data. In the target process the specific nutrition-related assessment data are documented in the "**ICF-Dietetics Assessment Sheet**" (Figure S1), which was adapted for dietetic use on the basis of the template from ICF Research Branch [53,54]. This is a document that can contain both administrative and medical patient data and should also be integrated in the HIS. The assessment sheet can be adapted depending on the requirements and local conditions, such as the hospital's corporate identity, technical formats or administrative guidelines. It serves to support the assessment in a structured and complete form [50,54].

The explicit differentiation of patient data between administrative and medical data in this model is based on structuring assessment information according to the bio-psycho-social model of the ICF [5].

The administrative data are required for both the environmental and the personal factors. Administrative patient data such as marital status and place of residence can be found in the respective electronic health record (EHR). Social context factors as defined by the ICF are currently not included in the EHR. If necessary, such data and administrative patient data that is missing for the dietetics assessment, must be determined by the dietitian. The medical patient data, such as laboratory data and medical diagnosis (classified by International Classification of Diseases (ICD) [7]), can be viewed in the HIS. All examinations and diagnostic findings carried out in the hospital are standardly displayed in the HIS. Additional findings provided by the patient can be scanned in by the hospital administration when the patient is admitted and thus be available electronically. Findings brought along that have not yet been electronically integrated into the nutritional survey and are only available in paper form can be viewed by the dietitian on site. If required medical data cannot be provided, they can be collected from the patient by the dietitian during the assessment. Examples of this are blood pressure values or the calculation of the body mass index (BMI).

The medical diagnosis and the patient ID should be entered automatically when the document is activated in the HIS. The line entitled “patient perspective” records the patient's view of their problems. It contains information about the patient's nutrition-related problem and which aspects he would like to change or achieve as free text. The further area “dietitian perspective” is divided into the ICF components “body structures/body function”, “activities” and “participation”. In this area, all information is taken from the documentation of other professional groups, e.g., existing medical data, weight, height and the BMI calculated from it, including the screening or test results as well as all results of the assessment by the dietitian, e.g., results of the target-actual comparison. The data for the contextual factors as “environmental factors” and “personal factors” must be completed in the last lines. The structuring of the assessment information in the assessment sheet helps the dietitian to recognize interactions between the components of the bio-psycho-social model of the ICF. This moves on to the process of identifying the nutrition-related problem and formulating the dietetics diagnosis.

Proposals for improving the “ICF-Dietetics Assessment Sheet”

The "ICF-Dietetics Assessment Sheet" (Figure S1) serves to support the documentation of the dietetics assessment according to the bio-psycho-social model of the ICF. It has a clear structure and enables the structured implementation of data collection and assessment of the nutritional status. However, structuring according to the components of ICF-Dietetics to determine the (functional) state of health has the disadvantages of flexibility and documentation support: in dietetics assessment, data

from other areas of the EHR, e.g., weight, height and BMI are required. This data can be automatically integrated and displayed in the document as a fixed component and would not have to be entered by the dietitian. In the current form of the dietetics assessment sheet, however, these (structural) fields are not shown or there are no separate areas for them. The components of the perspective of dietitians are presented as free text in the current template. According to the process specification, the ICF codes should be displayed in the dietetics assessment sheet in the future. The required fields (codes and titles) must be included in the template so that they can be taken from the ICF-Dietetics catalogue. Moreover, it should be possible in future to transfer the ICF codes and titles saved in the assessment sheet to further documents. In addition, the assessment should be carried out with dietetics core sets (predefined most important ICF-Dietetics categories) that are assigned to the respective medical diagnosis or setting. The "ICF-Dietetics Assessment Sheet" requires the fields for the transmission of planned core set data. These are not available in the current template. An alternative would be a link to the core sets to be used or a list of the core sets on the assessment sheet. This would support inter- and interprofessional communication and increase the transparency of the documentation.

The medical diagnoses are transferred to the assessment sheet. At the moment, however, it is not clear what type of diagnosis (admission diagnosis, referral diagnosis, main hospital diagnosis) it is, or whether there are other diagnoses that may not have been taken into account. A reference to the type of diagnosis, e.g., with an "[A]" for admission diagnosis could help to clarify the situation. Due to the large number of hospital diagnoses of a patient, it must be clarified whether all diagnoses or only those diagnoses relevant to the service request are shown. Here it must be determined who carries out the selection of the diagnoses to be documented. It should also be clarified whether the medical diagnosis code according to the ICD is also displayed in order to facilitate evaluations. The "ICF-Dietetics Goal Setting Sheet" (Figure S3) also contains the medical diagnosis. In this regard, the same aspects apply as for the "ICF-Dietetics Assessment Sheet".

**ID Number:**<br>**Med. Diagnose:**			
**Patient Perspective**			
	**Body structures /Body functions**	**Activities**	**Participation**
**Dietitian Perspective**			
	**Environmental factors**	**Personal factors**	

Figure S1: Document template "ICF-Dietetics Assessment Sheet" adopted from [53,54].

Future perspective

In future, it is planned that the most important categories (core sets for dietetics) for the respective disease or diagnosis, respectively will be proposed to the dietitian when entering the medical diagnosis or the medical referral issue for a standardized assessment. In addition, the assessment of information, such as the actual-target comparison of anthropometric and laboratory data, as well as the supply of energy and nutrients, should be supported by information technology. It is planned to include the ICF-Dietetics categories already in the assessment.

2. Dietetics Diagnosis

The purpose of the dietetics diagnosis is to identify a prioritized nutrition-related problem and to describe it, taking into account the cause(s) and symptoms and signs as well as facilitators and barriers [62]. To this end, the dietitian must first identify one or more nutrition-related problems (P) based on the assessment information. These nutrition-related problems should be classified with a classification catalogue. Further the diagnosis formulation includes the identification of the etiology (E) and the symptoms or signs (S) of the problem and according to the ICF coding guidelines the facilitators (F) and barriers (B) [5]. A dietetics diagnosis is made from these five criteria. This combination of criteria results in the PES-FB statement of the dietetics diagnosis. An example is shown in Figure S2. The prioritized dietetics diagnosis is then entered on the document **"ICF-Dietetics Goal Setting Sheet"** (Figure S3). In the right column, there is the area for the medical diagnosis as well as the prioritized dietetics diagnosis. With the entry of the dietetics diagnosis in the "ICF-Dietetics Goal Setting Sheet", the process of the dietetic diagnosis ends and the dietetics goal setting starts.

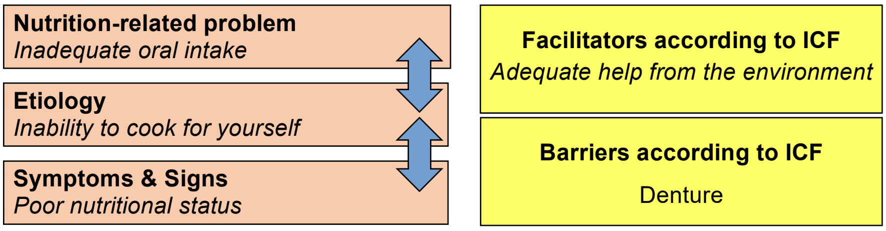

Figure S2: Criteria for the dietetics diagnosis statement (PES-FB) with an example translated from [63].

Proposals for improving the "ICF-Dietetics Assessment Sheet" for documenting the dietetics diagnosis

The "ICF-Dietetics Assessment Sheet" is used to illustrate the nutrition-related problem. The documentation of the PES-FB statement according to the target process must be added as free text. The documentation of the prioritized dietetics diagnosis in the goal setting sheet alone is viewed as inadequate, since further dietetics diagnoses can be made and must therefore also be documented. If only one of several diagnoses is shown in the subsequent document, the assessment sheet must be activated for inspection for all further findings or diagnoses. The illustration of the prioritized dietetics diagnosis in the "ICF-Dietetics Goal Setting Sheet" should contain a reference to the associated components of the assessment sheet so that the diagnosis can be traced.

Future perspective

In the future, there should be a separate catalogue for each diagnostic criterion, the criteria of which are linked to one another. The possible combinations of these criteria should subsequently be specified in a separate catalogue so that the diagnosis can be supported by medical expert systems in the future. The aim is to automatically determine one or more combinations according to the "nutrition-related problem catalogues" from the data obtained on the assessment sheet in order to subsequently determine the prioritized dietetics diagnosis. The dietetics specialist knowledge transferred into the medical expert systems for making diagnoses will be based on evidence-based guidelines and calculations. The algorithms and the necessary data are not yet specified in more detail.

All administrative and medical patient data, as well as those from the nutrition-related assessment, must be made available electronically before an automatic calculation by the expert system (e.g., energy and nutrient requirement calculation). Ideally, the values of the individual criteria calculated by the expert system are shown in the document "ICF-Dietetics Assessment Sheet" (Figure S1) and the prioritized dietetics diagnosis in the document "ICF-Dietetics Goal Setting Sheet" (Figure S3). The dietitian reviews the results and adds, removes or corrects the data if necessary.

3. Dietetics Goal Setting

After the dietetics diagnosis has been formulated, the dietetics goals are determined based on this. The **"ICF-Dietetics Goal Setting Sheet"** (Figure S3) is used to document the dietetics goal setting. The number of lines (ICF dietetic codes) is not limited.

The dietetics therapy goal is a long-term goal of the overall therapy. The intervention goal focuses on improving or solving the nutrition-related problem of the prioritized dietetics diagnosis. It refers to the comprehensive dietetics care, the elimination of a problem on a meta level [62]. Dietetics action goals and the patient's goals are sub-goals of the intervention goals and relate to them. The patient's goals are developed during the consultation by the patient or in agreement with the patient and represent the goals for discharge [62].

The intervention goals should be adopted using a catalogue of goals based on the corresponding nutrition-related problems. Based on the goals and other data from the dietetics assessment, the functional state of health of the patient is coded according to ICF [5]. However, currently, neutral ICF-Dietetics codes and categories are used to classify body functions, body structures, activities and participation. Instead of the qualifiers according to the ICF [5], the initial values are recorded and goal values are set for each ICF-Dietetics category (indicator). The number of goal values is adapted to the respective monitoring frequency. The initial values or situation must be completed by documenting the measuring instruments or sources used [62]. The "environmental factors" and "personal factors" are to be documented separately with the ICF qualifiers. In the lines for the respective ICF codes, the subjective assessment of the current situation must be entered as "facilitators" or "barriers" in accordance with the ICF guidelines [62].

**1 Long term goal:**		**Med. Diagnosis:**<br>**Dietetics Diagnose:**			
**2 Intervention goal:**					
**Action goal:**					
**Action goal:**					
**Action goal:**					
**Action goal:**					
**Patient goal:**		**Patient goal:**			
**Patient goal:**		**Patient goal:**			
**ICF Code**	**Body functions/Body structures**	**Assessment**		**Goal value 1**	**Goal value 2**
		**Description / Initial value**	**Instrument /Measurement**	until:	until:
**ICF Code**	**Activities/Participation**				
**ICF Code**	**Environmental factors**	**facilitator**<br>+4 +3 +2 +1	**barrier**<br>1 2 3 4		
**ICF Code**	**Personal factors**	**facilitator**<br>+4 +3 +2 +1	**barrier**<br>1 2 3 4		

Figure S3: Document template "ICF-Dietetics Goal Setting Sheet" adapted from [53,54].

Proposals for improving the "ICF-Dietetics Goal Setting Sheet"

For a better overview and communication, free text shout be integrated into the dietetics goal catalogue as suggestion texts. An alternative is the free text documentation in a separate field. Furthermore, with regard to goal setting, it should be take into consideration that the ICF-Dietetics codes and categories are shown for the first time in the "ICF-Dietetics Goal Setting Sheet", although they have already been determined in the assessment. Thus, for the processing of the goal setting sheet, the view of the completed assessment sheet is required. This can be avoided by creating and displaying relevant indicators for goal setting in the assessment sheet with automatic transfer to the goal setting sheet. In regard of the goal setting sheet, it also has to be mentioned that different types of goals (therapy, intervention, action and patient goals) are defined in the document. However, it is not clear from the document which (time-dependent) action goal is referred to which time goal value. Labelling could be done by adding the number of the action goal to the target value, e.g., "[H1]".

Future perspective

In the future, if the ICF-Dietetics codes are used in the assessment sheet, relevant ones for the goal setting can be taken over by this. An ICF-Dietetics subject heading catalogue to locate ICF-Dietetics codes and assisted by automatic coding is another step considered.

The support from expert systems in the goal setting process should include the assignment of the ICF-Dietetics codes to the goals as well as automated target-actual comparison calculations. The up-to-date version of the guidelines and reference values must be available.

The ICF-Dietetics codes are determined from the free texts of the respective parts of the assessment sheet. This should be done largely by mapping the texts to the associated codes. The calculation of values in the initial and goal values should automatically result from a data table to be specified by algorithms, for example the weighed weight as the initial value and the “normal” weight from a table. The calculation of the proposed goal value "weight" at a point in time is calculated from this data, among others. The calculated values are to be regarded as a suggestion or a basis for discussion. The assigned codes and values are checked by the dietitian and adapted if necessary.

4. Dietetics Intervention

After the “ICF-Dietetics Goal Setting” document has been completed, the dietetics intervention takes place on this basis. The ICHI-Dietetics will serve as standardized terminology for documentation interventions in the future. Therefore a separate document will be provided.

Future perspective

The selection of interventions with support of an expert system is part of further considerations. Based on the dietetics diagnosis and the goal values from the goal setting sheet, the respective interventions should be automatically proposed and recoded to the ICHI-Dietetics Catalogue. A manual processing and overriding of the selected interventions by the dietitian should be possible.

5. Dietetics Evaluation

The last part in the DCP of the target process concerns the evaluation of the previously set goals. The implementation takes place through systematic comparisons of the current status with the previous status as well as the comparison of the goal values with the current values. The results of the evaluation are entered in the **“ICF-Dietetics Evaluation Sheet”** (Figure S4). The data are taken from the “ICF-Dietetics Goal Setting Sheet” (Figure S3). For all other goal values, proceed in the same way as the first evaluation in a new column.

Future perspective

Expert systems should support the dietitian by calculating current values (e.g., BMI) and automatically entering them in the field provided. An integrated automatic assessment of the patient's goals in the document (e.g., achieved/not achieved/partially achieved) by means of textual or color coding should be considered.

The documentation of the DCP ends with the entry of the last evaluation values (outcomes evaluation). Finally, evaluations can be carried out to improve the quality of the sub-processes and the overall process. Own documents in this regard are provided as a checklist [62].

1 Long term goal:		Med. Diagnosis:			
2 Intervention goal:		Dietetics Diagnose:			
Action goal:					
Action goal:					
Action goal:					
Action goal:					
Patient goal:		Patient goal:			
Patient goal:		Patient goal:			
**ICF Code**	**Body functions/Body structures**	**Assessment**		**Evaluation**	
		**Description / Initial value**	**Instrument/Measurement**	Date: Goal value	Eval. value
**ICF Code**	**Activities/Participation**				
**ICF Code**	**Environmental factors**	**facilitator** +4 +3 +2 +1	**barrier** 1 2 3 4		
**ICF Code**	**Personal factors**	**facilitator** +4 +3 +2 +1	**barrier** 1 2 3 4		

Figure S4: Document template "ICF-Dietetics Evaluation Sheet" adapted by [53,54].

General considerations in regard to medical expert systems

Currently, neither medical expert systems to assist dietitians in performing the DCP nor projects to create medical expert systems in the field of dietetics are known. Possible future integration options were considered in the analysis of the target process, however an assessment of the integration of medical expert systems is not possible. On one side, due to the complexity of expert systems and on the other side due to missing prerequisites for the calculations of the medical expert systems. Up-to-date guidelines and treatment standards are required not only for the dietetics intervention, but also for other sub-processes. For example, it requires validated disease- or setting-specific core sets for the dietetics assessment. Equally important are validated nutrition-related problems and dietetics diagnosis in relation to important nutrition-relevant medical diagnosis (with ICD coding). Furthermore, that expert systems can be used effectively at the time of the calculation, e.g., BMI, all data in the HIS must be available in a usable format. In any case, support from expert systems makes sense, as it leads to savings in working time and quality improvements and should therefore be considered further.
