# Supplementary material for: Integrating a New Dietetic Care Process in a Health Information System: A System and Process Analysis and Assessment
Source: Int J Environ Res Public Health. 2022 Feb 22;19(5):2491. doi: 10.3390/ijerph19052491 (PMC8909013; doi:10.3390/ijerph19052491)
Supplement: Supplementary file 1 [file ijerph-19-02491-s001.zip › ijerph-1533807-supplementary/Supplementary Material S2.pdf]

Integrating a new dietetic care process (DCP) in a health information system (HIS): a system and process analysis and assessment

Gabriele Gäbler, Deborah Lycett and Walter Gall

Description of the current process and adaption of the new DCP

The current process was visualized as business process model (Table S1 shows the notations according to [64,65]) and is divided into four sub-processes (“dietological service request”, “service request analysis”, “dietological visit” and “dietological documentation”). After the “dietological service request” (Figure S5) has been transferred to the dietitian, the sub-process “service request analysis” (Figure S6) is initiated by the dietitian. Finishing of the analysis triggers the sub-process of the “dietological visit” (Figure S7) at the patient. The subsequent sub-process “dietological documentation” (Figure S8) finalises the current process.

The representation of these sub-processes by means of eEPC takes place as process interfaces. Due to traceability and better orientation for the system and process technology assessment, the associated sub-process of the new DCP with the associated documents is shown to the right of the eEPC functions (Figure S6 to S8). The current sub-process “dietological service request” (Figure S5) has no corresponding part in the new DCP, as it is not carried out by dietitians. The current sub-process "service request analysis" (Figure S6) includes the beginning of the dietetics assessment of the DCP parallel to the step “analyse patient data". The "ICF-Dietetics Assessment Sheet" (Supplementary Material S1) can be used here by already entering data from the electronic health record (EHR) into the dietetics perspective or by taking notes for the patient visit. Furthermore, data on the nutritional status (e.g., weight) can be transferred from the EHR. The “dietological visit” (Figure S7) begins with the task “take anamnesis (history)”, which is also assigned to the dietetics assessment of the DCP. The "ICF-Dietetics Assessment Sheet" can also be used here. The task "make preliminary finding" corresponds to the dietetics diagnosis and is documented with the "ICF-Dietetics Goal Setting Sheet" (Supplementary Material S1). The task “define goals” in the current process corresponds to the dietetics goal setting of the DCP and is also documented with the “ICF-Dietetics Goal Setting Sheet”. The task “clarify interventions” of the current process is equivalent with the dietetics intervention of the DCP and is documented with the “ICHI-Dietetics Intervention Sheet” (currently not available). The last task of the sub-process “dietological visit” “clarify final questions” includes part of the dietetics evaluation of the DCP. The current sub-process "dietological documentation" (Figure S8) initially includes the administrative performance documentation and the finding documentation. This finding documentation contains all important findings of the “dietological visit”.

In contrast, the report from the new DCP is contained in several documents and carried out immediately, so the implementation of the documentation is in principle brought forward. If the dietitian does not create the new documents until this point in time, the task “create finding documentation” includes the creation of the “ICF-Dietetics Assessment Sheet”, “ICF-Dietetics Goal Setting Sheet” and the “ICHI-Dietetics Intervention Sheet”. The functions “determine follow-up appointment” and “enter follow-up appointment” are also part of the dietetics evaluation of the DCP.

The use of the “ICF-Dietetics Evaluation Sheet” (Supplementary Material S1) is not required at this time point, as only the appointment reservation and not the evaluation is carried out. The new DCP requires adaptations for the evaluation as an additional task and an additional document the “ICF-Dietetics Evaluation Sheet”.

Table S1: Extended event-driven process chain (eEPC) notations according to [64, 65].

**Component**	**Symbol**	**Description**
Process Interface	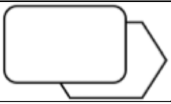	Linking to upstream or downstream business processes
Data object	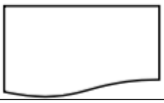	Incoming and outgoing documents for a function
Function	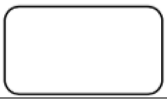	Transformation of a status into a target status (time-consuming element)
Event	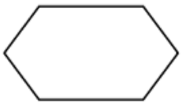	Time related status (element with no time-consumption); It triggers a function or is a result of a function
AND	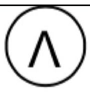	AND Connector: logical AND
XOR	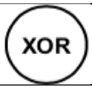	OR Connector: logical exclusive OR
Control Flow	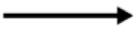	Shows the flow direction
Assignment		Assignment of one element to another
Database	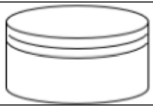	Storage with direct access
Organizational Element	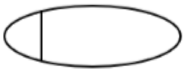	Organization unit, e.g., Department, Division, etc.
Informational Object	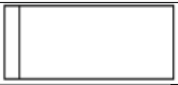	Role of a person
Informational Object	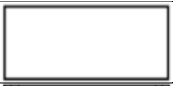	Location of function execution
Informational Object	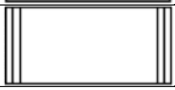	Any kind of Information, information carrier or information system

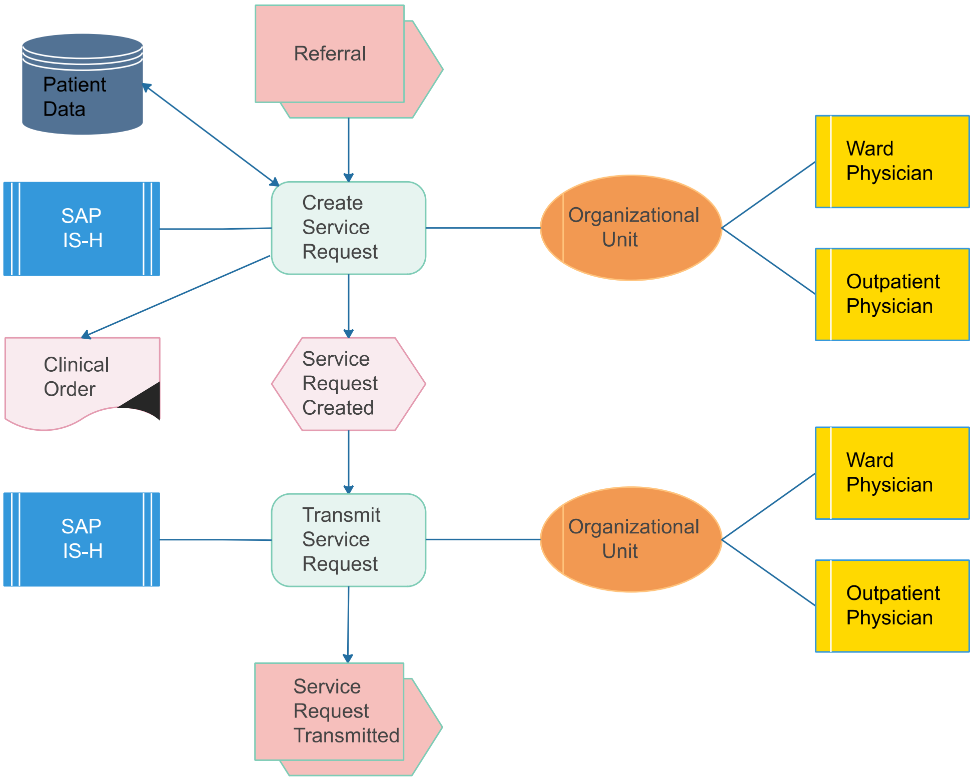

Figure S5: Current sub-process "Dietological Service Request" (adapted from Krottenmüller [63]).

At the beginning of the process there is the process interface "referral" due to a need within the overall medical process. This request triggers the associated function "create service request". The request is made by a physician on a ward or an outpatient department. The service request is created in SAP "IS-H" [68]. The physician is provided with the necessary patient data from the database in the service request mask by means of the SAP "IS-H" system. In terms of system technology, the "clinical order" (KLAT) (The KLAT is an SAP-specific terminology that represents a requirement that is imaged in the system. It is used to order treatments or to reserve patients for treatment. The term "clinical order" can be used synonymously.) document is generated. The expression "dietological council" is not a clinical order type and does not have to be entered as a title, but results from the free text question asked by the physician. The service request is created by saving the document. The clinical order is then. This is done by changing the document status to "release". The system transmits the KLAT to the service provider. The SAP "IS-H" system is used for communication. The sub-process "dietological service request" ends with transmission of the KLAT.

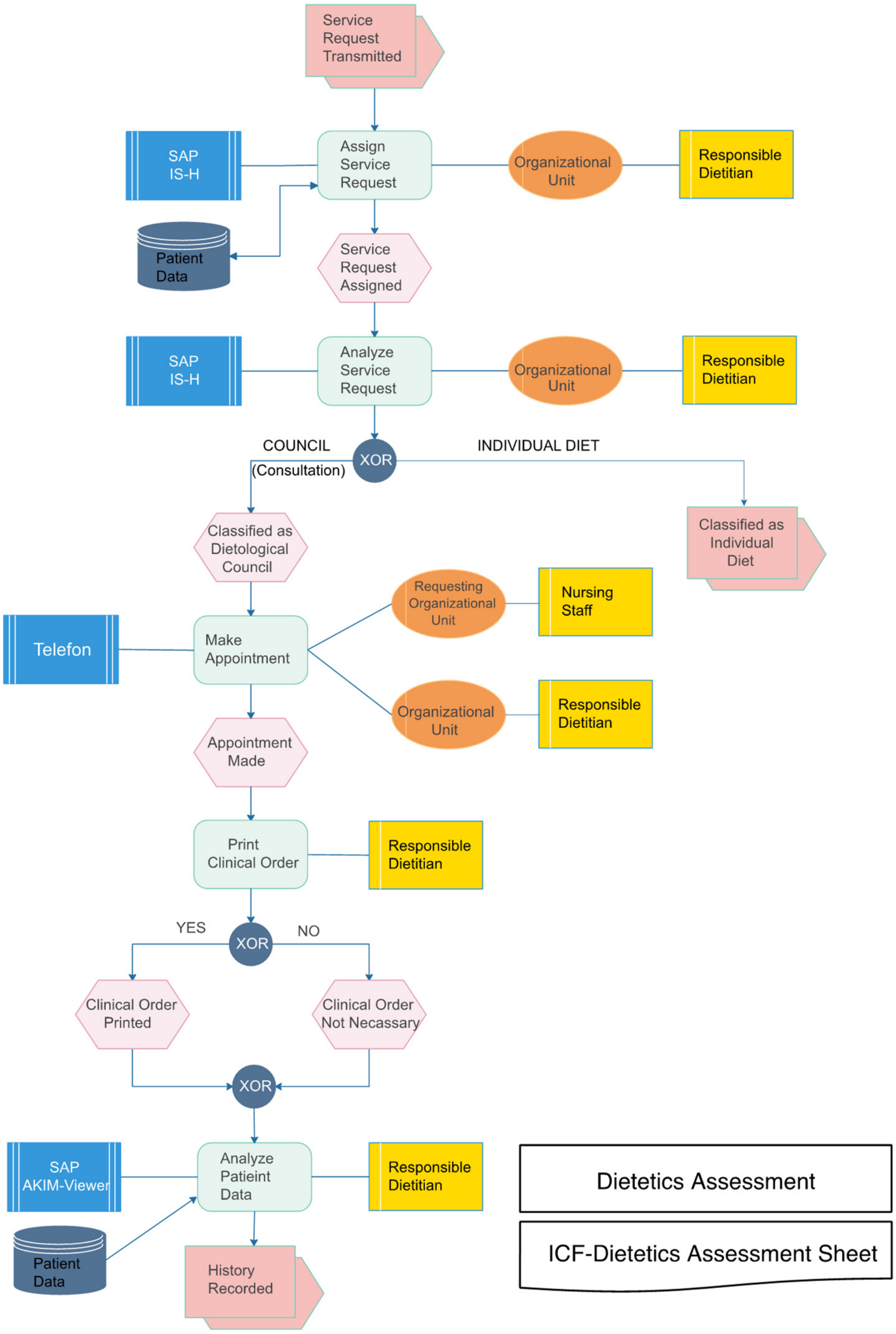

Figure S6: Current sub-process "Service Request Analysis" (adapted from Krottenmüller [63]).

The event "service request transmitted" starts the sub-process "service request analysis". The dietitian responsible for processing, assigns the "service request" in the SAP system to the dietitian responsible for applying the dietetic expertise. In terms of system technology, this is done by entering the dietitian as the operator of the service. With the entry, the responsible dietitian receives the service in his personalized SAP "IS-H" area of the clinical workstation. This entry causes a change in the patient master data. The assigned dietitian analyses the content of the "service request". A separate order must be created for each individual service. Due to the technical system setting, only one service per KLAT is explicitly requested. The request can either be a "dietological council" or an "individual diet" for the patient. The request "individual diet" is not subject of this analysis and is therefore defined as a process interface. In the case of a request "dietological council", an appointment with the patient for an on-site dietological visit is required, that is made by telephone with the ward or outpatient department. Any questions that arise from the "service request" are also clarified per phone. If necessary, the KLAT "clinical order" is then printed out to support the anamnesis or it is not necessary. The patient data of the HIS is accessed with the SAP tool AKIM-Viewer. The dietitian analyses the EHR, necessary administrative data (e.g., marital status) and, if necessary, additional findings available in the SAP system. Reviewing patient data does not require any data changes. After analysing patient data, the process ends with the event "dietological history recorded".

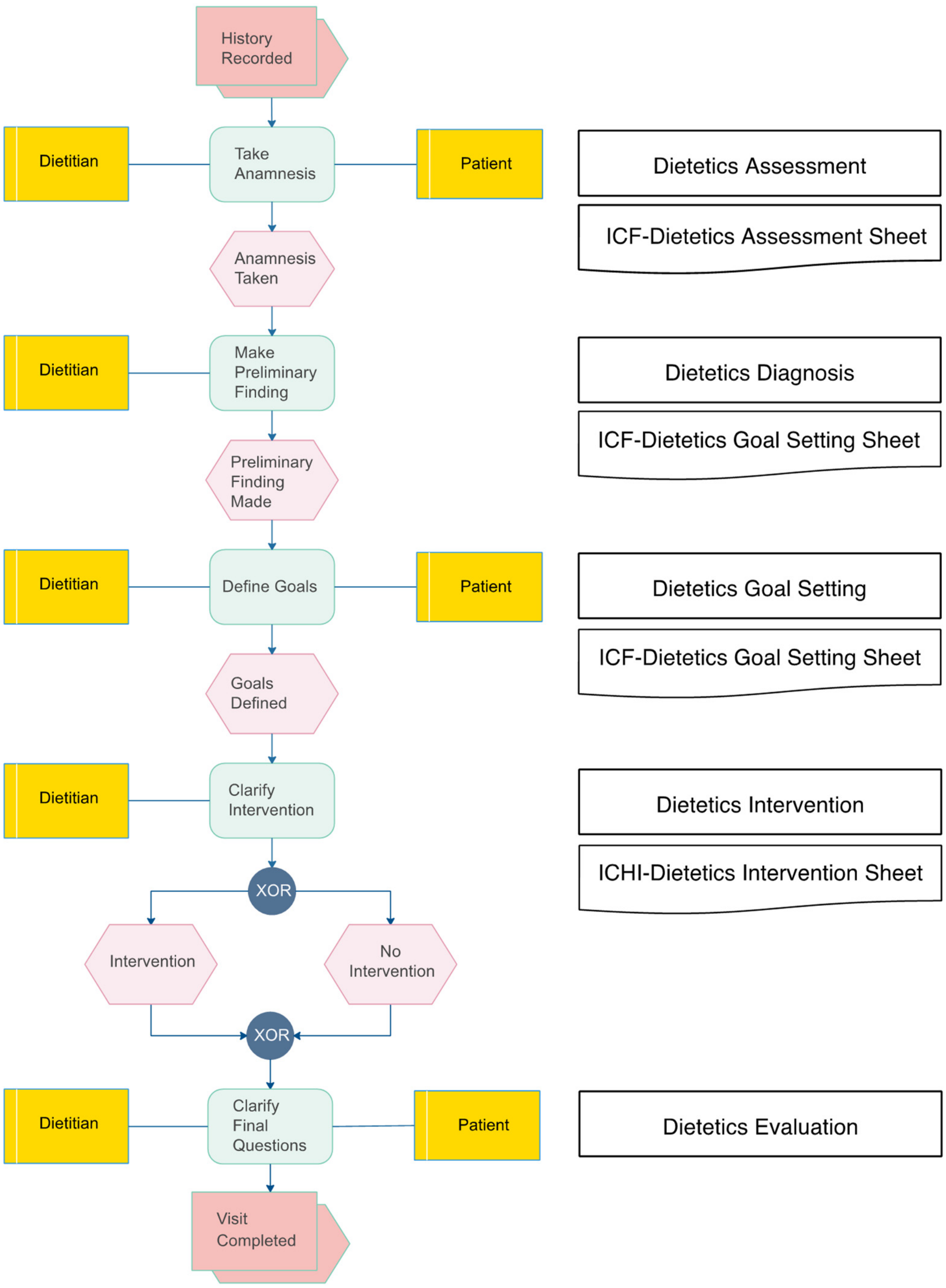

Figure S7: Current Sub-process "Dietological Visit" (adapted from Krottenmüller [63]).

With the event "dietological history recorded", the sub-process of the "dietological visit" begins with the task "take anamnesis". Which is carried out on the ward or outpatient department. During the anamnesis, other patient data not visible in the EHR, such as height and weight requested to calculate the BMI. For the anamnesis, the dietitian uses their own anamnesis templates, which list the most important information to be carried out. The templates are different depending on the requirement and the dietitian. For example, the gastroenterological anamnesis contains as a minimum the medical diagnoses, a vegetative anamnesis, the nutritional status, laboratory, medication, social anamnesis, nutritional anamnesis (24-hour recall), nutrient requirement calculation and target-actual comparison and interpretation. As soon as the anamnesis is finished a preliminary dietetic finding is made on site. The subsequent definition of goals takes place together with the patient. After the goals has been defined, possible interventions are discussed with the patient and, if necessary, perform afterwards. The "dietological visit" is completed with the clarification of final questions with the patient, e.g., further visits. The HIS is not used directly during this process.

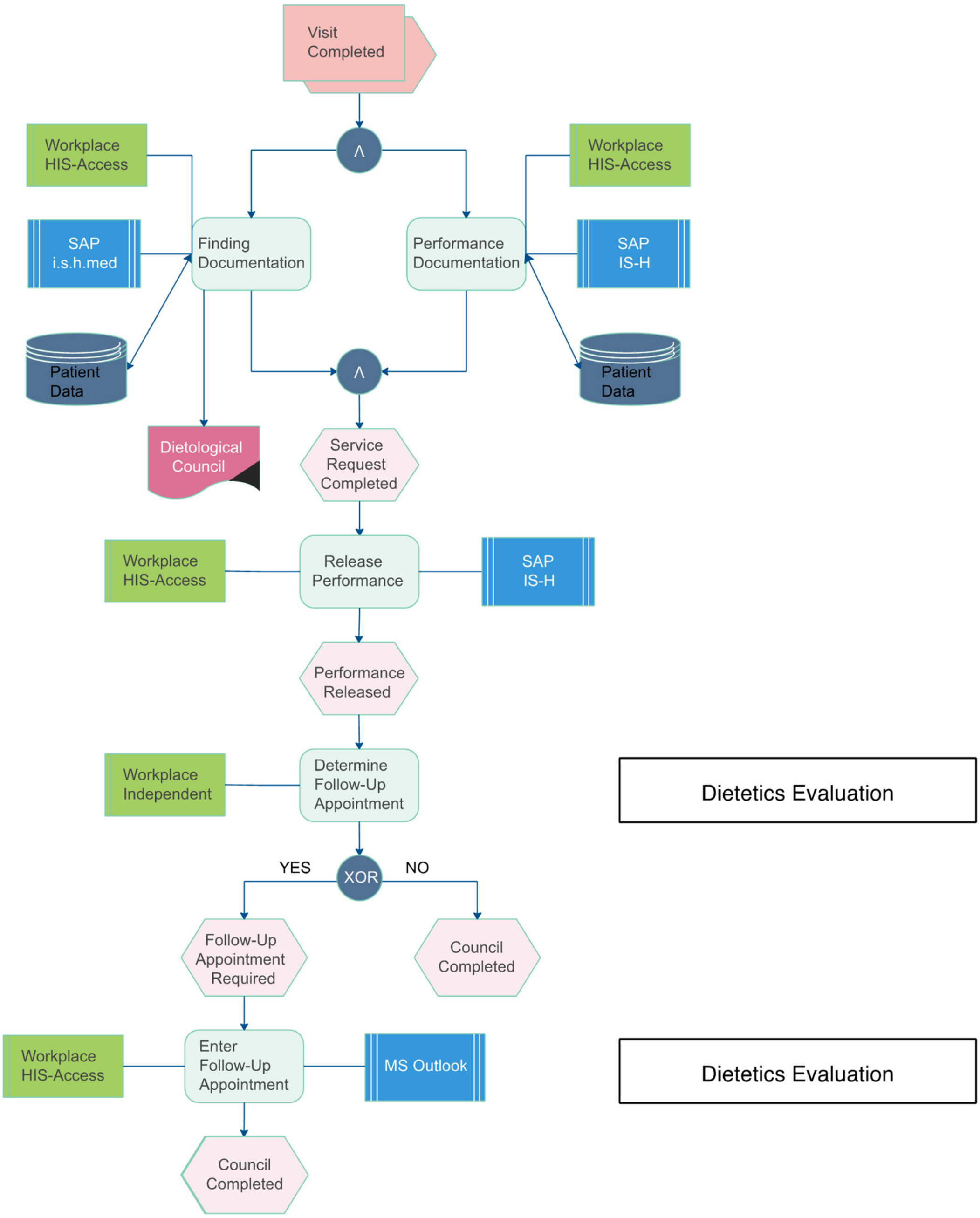

Figure S8: Current sub-process „Dietological Documentation" (adapted from Krottenmüller [63]).

The sub-process "dietological documentation" begins after completing the "dietological visit". The documentation process is divided into finding documentation and performance documentation. It is carried out entirely by the dietitian without the involvement of third parties. Both activities are entered in the SAP system and result in a database change in the EHR. By creating the "dietological council" as a separate SAP document, the EHR is automatically supplemented by this document. The current document contains the medical referral issue, a possible administrative comment (e.g., interpreting required), the performed dietetics intervention and actions (including performance codes), the dietological free-text diagnosis (finding) with vegetative anamnesis, nutritional status, laboratory, medication as well as social and nutritional anamnesis. Additionally, there is an interpretation of findings, a therapy recommendation, and the further procedure (e.g., follow-up). The focus of the consultation can be specified. Thus, physicians have access to the EHR, it is not necessary to actively send the document or notify that it has been created. The generated document is after approval automatically visible. The dietitian also enters the service documentation in the SAP "IS-H" module, which is used for the internal allocation of costs. The relevant individual services (e.g., "dietological advice") are selected from the internal service catalogue. Access to the HIS or SAP is a prerequisite for performing both activities. The dietitian can carry out the written diagnosis and service documentation on a computer with SAP access directly on the ward or outpatient department. After completing both activities, the performance can be released for internal billing. By changing the status of the service in SAP to

“performance released”, indicates that the “dietological council” has been finished and released. The “service request” is also no longer visible in the screen mask of the clinical workstation. Finally, if necessary, the responsible dietitian enters a follow-up appointment in outlook appointment calendar for evaluation of the intervention. This completes the process “dietological council”.
